# Association of Admission Blood Glucose Level with Major Adverse Cardiac Events in Acute Coronary Syndrome; a Cohort Study

**Published:** 2019-04-16

**Authors:** Mostafa Alavi-Moghaddam, Mohamad parsa-Mahjoob, Robabeh Ghodssi-ghassemabadi, Bita Bitazar

**Affiliations:** 1 Emergency Medicine Department, Imam Hossein Hospital, Shahid Beheshti University of Medical Sciences, Tehran, Iran. mosalavi@sbmu.ac.ir; 2 Cardiovascular Research Center, School of Medicine, Shahid Beheshti University of Medical Sciences, Tehran, Iran.; 3 Department of Biostatistics, Faculty of Medical Sciences, Tarbiat Modares University, Tehran, Iran.

**Keywords:** Blood glucose, acute coronary syndrome, myocardial infarction, stroke, death

## Abstract

**Introduction:**

Appropriate management of abnormal admission blood glucose level (ABGL) in acute coronary syndrome (ACS) patients still remains a common issue. This study aims to assess the influence of ABGL on development of 30-day major adverse cardiac events (MACEs) in patients with suspected ACS.

**Methods:**

This is a prospective cohort study based on analysis of data collected from patients suspected to acute coronary syndrome admitted to emergency department. ABGL of patients was measured and its association with development of MACEs (MI, CVA, mortality) within 30 days of follow-up was studied.

**Results:**

814 participants with the mean age of 61.8 ± 13.4 years were studied (58.1% male). MACE endpoints were developed in 166 (39.0%) hyperglycemic, 30 (46.9%) hypoglycemic, and 53 (16.4%) normoglycemic patients (p<0.001). Mean admission blood glucose level of patients who developed MACE within 30 days was significantly higher than others (210.6 ± 123.4 vs 157.4 ± 86.6mg/dL; p<0.001; OR: 1.006 (1.005 to 1.008)). There was a significant correlation between male gender (p=0.027), abnormal admission blood glucose level (p<0.001), diabetes (p = 0.001), hyoerlipidemia (p=0.059), prior CABG (p=0.008), first and second blood troponin levels (p<0.001), first and second abnormal ECGs (p<0.001), and also ECG changes (p<0.001) with developing MACE. Abnormal ABGL, first and second blood troponin levels, and the history of diabetes were among independent risk factors of developing MACE within 30 days.

**Conclusion:**

It seems that abnormal admission blood glucose level in suspected ACS patients was an independent predictor of major adverse cardiac events within 30 days.

## Introduction:

Associations between elevated admission blood glucose level (ABGL) and bigger infarct size in acute myocardial infarction (AMI) as well as inflammation in acute coronary syndrome (ACS) have been reported (1, 2). Many studies have argued about the possible direct impact of hyperglycemia on adverse outcomes of acute coronary syndrome patients through various pathophysiological mechanisms. Recent studies have suggested that hyperglycemia has a detrimental effect on ischemic myocardium. It has been reported that acute hyperglycemia abolishes ischemic preconditioning and promotes apoptosis (3, 4). Acute hyperglycemia also decreases nitric oxide bioavailability, impairs endothelial function, increases platelet aggregability and stimulates coagulation (5). These changes may cause microvascular dysfunction during reperfusion and impaired left ventricular function after AMI (6). 

This theory has a measure of support from studies that have identified a stronger correlation between the risk of ACS in patients with hyperglycemia without a history of diabetes (7). There is a biological plausibility to this result as these patients may have an undiagnosed, untreated diabetic state resulting in more glycolytic damage than someone known to have diabetes and actively receiving treatment. Similar findings were obtained by Petursson et al. when assessing 30-day mortality risk of patients with confirmed ACS (8). This result has also been reported in patients experiencing AMI (9). 

Some studies have suggested insulin administration in patients with severe hyperglycemia at the time of admission in emergency department regardless of their diabetic state (10, 11). In a guideline published by National Health System of the Great Britain, insulin administration is recommended in patients with confirmed ACS with admission blood glucose level higher than 198 mg/dl (12). In a Japanese study, although they stated that hyperglycemia was observed in many patients with ACS, they emphasized that administrating insulin requires more researches (13).

A number of large therapeutic studies have attempted to explain the effect of hyperglycemia on post-ACS mortality, but the lack of a general consensus on the appropriate management strategy for abnormal glycemia still remains a common issue in emergency departments. In addition, adverse outcomes risk stratification in cardiac patients and early prediction of major adverse cardiac events (MACE) is a matter of importance. Based on above-mentioned points, this study aims to assess the influence of admission blood glucose level (ABGL) on development of 30-day MACE in patients with suspected ACS.

## Methods:

***Study design and setting***

This is a prospective cohort study based on analysis of data collected from patients with suspected acute coronary syndrome admitted to emergency department of Imam-Hossein Hospital, Tehran, Iran, between June 21, 2016 and June 20, 2017. Presenting blood glucose level of patients was measured and its association with development of MACE within 30 days of follow-up was studied. The protocol of this study was approved by Ethics committee of Shahid Beheshti University of Medical Sciences (number: IR.SBMU.RETECH.REC.1397.519) and researchers adhered to principals of Helsinki protocol and confidentiality of patients’ information. Informed consent was obtained from patients or his/her relatives before enrollment to the study. 

***Participants***

Patients were included if they fulfilled all of the following criteria: ≥18 years of age; ≥5 minutes of symptoms suggestive of ACS; and the attending physician deciding to investigate with cardiac biomarkers. Patients with a clear cause other than suspected ACS for the symptoms (e.g. clinical finding of pancreatitis), transfer from another hospital, pregnancy, previous enrollment, and inability to be contacted after discharge were excluded. 

The American Heart Association (AHA) case definitions for symptoms suggestive of a cardiac condition were used, which include chest pain, epigastric, jaw or arm pain, or discomfort or pressure without an apparent non-cardiac source.

***Measurements and outcome***

After careful history taking, physical examination, and doing initial assessments, eligible patients were selected. Non-fasting, on-admission blood glucose level was measured for all patients. Blood samples for measuring blood glucose were taken from patients’ finger tips. All measurements were done by the same glucometer and the same glucose test tape.

All patients were evaluated regarding the development of MACE within the 30 days of follow-up. Acute myocardial infarction (AMI), cerebrovascular accident (CVA), and all-cause mortality were considered as MACE in this study. Patients’ follow-up was done by a senior emergency medicine resident via phone calls. 

***Data gathering***

The baseline characteristics (such as age and gender), medical history, contact information, and ECG and laboratory findings as well as 30-day outcome were recorded in special paper sheets. 

Blood glucose level ≤90 mg/dL was considered as hypoglycemia, 91 to 126 mg/dL as normalglycemia, and >126 mg/dL as hyperglycemia. All data were collected by a senior emergency medicine resident under direct supervision of an emergency medicine specialist. 

***Statistical analysis***

The statistical analyses were performed using R statistical (version 3.2.1) and SPSS 21 software. Continuous data were presented as mean and standard deviation, and categorical data were presented as frequency and percentage. Logistic regression analysis was done concerning the variables age, gender, hypertension, dyslipidemia, diabetes, family history of cardiac disease, smoking, prior AMI, prior CABG, cardiac troponin and ECG changes. The relationship between the three ABGL categories and MACE within 30 days was analyzed. Area under the ROC curve (AUC) was calculated in order to calculate predictive accuracy of HEART score and model containing HEART score and ABGL. Significance level was set at 0.05.

## Results:

***Baseline characteristics of studied patients***

**Figure 1:**
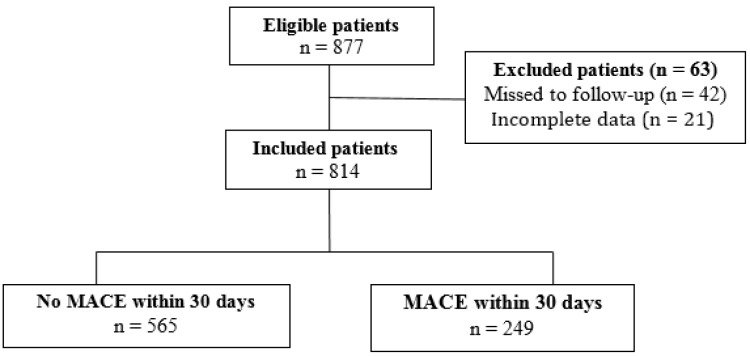
Flow diagram of participant recruitment

877 patients suspected to ACS were evaluated. 63 of whom were excluded (42 were missed to follow-up and 21 had incomplete data; figure 1). Finally, 814 participants with the mean age of 60.8 ±13.4 (23 – 91) years were entered to analysis (58.1% male). The mean admission blood glucose level of patients was 173.7±102.2 (71 – 540) mg/dL. 64 (7.9%) patients were hypoglycemic, 324 (39.8%) normoglycemic, and 426 (52.3%) hyperglycemic at the time of admission to emergency department. 

MACE endpoints were identified in 249 (30.6%) patients within the 30 days of follow-up (231 (28.4%) AMI, 12 (1.5%) CVA, and 62 (7.6%) death cases). Table 1 compared the baseline characteristics of studied patients based on the presence or absence of MACE. MACE endpoints were developed in 166 (39.0%) hyperglycemic, 30 (46.9%) hypoglycemic, and 53 (16.4%) normoglycemic patients (p<0.001). The mean admission blood glucose level of patients who developed MACE within 30 days was significantly higher than others (210.6±123.4 vs 157.4±86.6mg/dL; p<0.001; OR: 1.006 (1.005 to 1.008)). The HEART score of patients with MACE was significantly higher (p < 0.001).

***Correlations***

**Figure 2:**
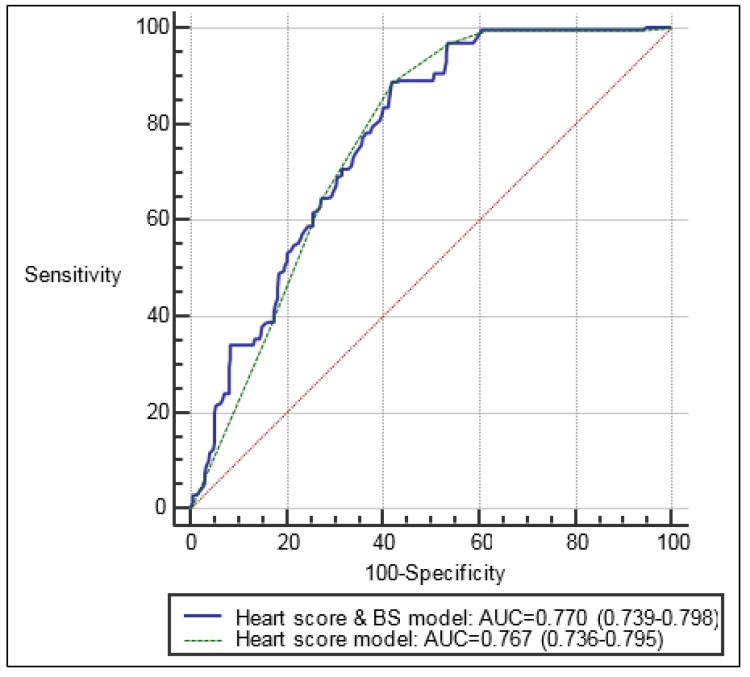
Area under the ROC curve of HEART score and a model containing HEART score plus blood glucose level in predicting the development of major adverse cardiac event (MACE) within 30-day follow-up of acute coronary syndrome patients

There were significant correlations between male gender (p=0.027), abnormal admission blood glucose level (p<0.001), diabetes (p = 0.001), hyperlipidemia (p=0.059), prior CABG (p=0.008), first and second blood troponin levels (p<0.001), first and second abnormal ECGs (p<0.001), and also ECG changes (p<0.001) with developing MACE. The results of multiple logistic regression model demonstrated that abnormal ABGL, first and second blood troponin levels, and the history of diabetes were among the independent risk factors of MACE within 30 days (Table 1). Figure 2 shows the area under the ROC curve of HEART score + ABGL and HEART score alone in predicting the development of MACE (0.770 vs. 0.767). 

## Discussion:

The present study has confirmed findings from previous reports that abnormal ABGL is associated with increased risk of developing MACE in ACS patients. The findings showed that ABGL could probably be an independent risk factor regardless of diabetic status or traditional risk factors of MACE; like a similar study done by Gardner et al. (14), where they demonstrated that within 30 days of follow-up, the odds of patients with ABGL higher than 7 mmol/L (126 mg/dL) developing MACE were 1.5 times higher than patients with an ABGL<7 mmol/L. Capes et al. [8] demonstrated that the relative risk of in-hospital mortality in non-diabetic MI patients with ABGL>6.1 mmol/L was 3.9 times higher than patients with normal glycemia. Among diabetic MI patients, those with ABGL≥10 mmol/L had a 70% increase in the risk of in-hospital mortality compared to normal glycemic diabetic patients. The largest retrospective study on this subject to date, which examined the outcomes of 141680 elderly patients with MI, demonstrated a significant 13-77% increase in 30-day mortality and a 7-46% increase in 1-year mortality depending on the degree of hyperglycemia (9).

**Table 1:** Baseline characteristics of studied patients based on development of major adverse cardiac events (MACE)

**P **	**OR (95% CI)** *	**P**	**MACE**		**Total**	**Characteristics**
			No n=565	Yes n=249		
**Sex**						
0.065	0.72 (0.51-1.01)	0.027	314 (66.4)	159 (33.6)	473	Male
			251 (73.6)	90 (26.4)	341	Female
						**Age**
> 0.05	1.01 (0.97-1.02)	0.395	60.6±13.4	61.5±13.4	60.83±13.40	Mean ± SD
**Blood glucose level (mg/dl)**						
<0.001	1.01 (1.01-1.01)	<0.001	157.4±86.6	210.6±123.4	173.7±102.2	On admission
**Comorbid disease**						
>0.05	1.36 (1.01-1.82)	0.144	300 (67.3)	146 (32.7)	446 (54.8)	Hypertension
	0.67 (0.43-1.04)	0.059	87 (77.0)	26 (23.0)	113 (13.9)	Hyperlipidemia
0.003	1.95 (1.43-2.64)	0.001	162 (61.8)	100 (38.2)	262 (32.2)	Diabetes
>0.05	1.23 (0.92-1.66)	0.586	229 (68.4)	106 (31.6)	335 (41.2)	Cardiac disease
	1.05 (0.71-1.56)	0.340	87 (65.9)	45 (34.1)	132 (16.2)	Smoking
	1.22 (0.69-2.15)	0.722	37 (67.3)	18 (32.7)	55 (6.8)	History of MI
	1.95 (1.24-3.08)	0.008	36 (43.4)	47 (56.6)	83 (10.2)	Prior CABG
**Positive troponin**						
0.025	2.91 (1.68-5.03)	<0.001	25 (43.9)	32 (56.1)	57 (7.0)	1^st^
0.004	2.93 (2.16-3.99)	<0.001	128 (48.3)	137 (51.7)	265 (32.6)	2^nd^
**ECG abnormality**						
> 0.05	1.95 (1.41-2.70)	<0.001	349 (64.6)	191 (35.4)	540 (66.3)	1^st^
	2.16 (1.55-3.01)	<0.001	349 (64.0)	196 (36.0)	545 (67.0)	2^nd^
**ECG changes **						
> 0.05	2.76 (2.04-3.72)	<0.001	162 (55.1)	132 (44.9)	294 (36.1)	Positive
**HEART score**						
<0.001	1.75 (1.59-1.92)	<0.001	7.88 ± 1.27	5.94 ± 2.32	6.54 ± 2.24	Mean ± SD

* unadjusted odds ration with 95% confidence interval (CI). Data are presented as mean ± standard deviation (SD) or number (%). MI: myocardial infarction; CABG: coronary artery bypass graft; ECG: electrocardiogram.

In the present study, the frequency of AMI endpoint was higher than CVA. This probably happened due to entering ACS patients to the study, most of which were affected with AMI. 

39.0% of hyperglycemic patients finally developed MACE within 30 days. Foo et al. (15) did a similar study in 3 east London hospitals over a 2-year period. They demonstrated a near-linear relationship between higher admission glucose levels and higher rates of cardiac death. Particularly in glucose groups, measures being near to or far from normal glycemia affected the risk of cardiac problems. Li Dong-bao et al. (16) demonstrated a U-shaped relationship between admission glycemia and in-hospital mortality in AMI patients, which means that hypoglycemic and hyperglycemic patients were high-risk, which matches findings of the present study.

In this study, elderly people and patients with a known history of diabetes and hyperlipidemia were more at risk. In Gardner et al. (14) study, the predictors of MACE in addition to admission glycemia were male gender, age, hypertension, ischemic ECG, and positive troponin, which matches the present study; but there was a mismatch in diabetes and hypertension variables. In a study in Poland, diabetes history, age, hypertension, and hypercholesterolemia had a significant relationship with MACE (17). The cause of non-identical results in various studies could be different sample sizes and the method of blood glucose assessment and study population classification.

It has been debated that whether hyperglycemia has a possible direct impact on adverse outcomes or is just a secondary factor. There is a hypothesis that proposes elevated blood glucose level is a marker of illness severity (18). It has been suggested that hyperglycemia is representative of an induced stress response proportional to the ischemic myocardial damage (14). This has been found in earlier studies where the size of an infarct was associated with a corresponding degree of creatine kinase MB, cortisol and catecholamine release and an associated linear increase in glucose (19). Therefore, it has been speculated that glucose may not necessarily be the causative agent leading to an increased risk of a MACE; but instead may simply act as a marker indicating the extent of myocardial damage, the presence of which is necessary for a MACE (20). 

If association of ABGl with developing MACE is proved, there will be hope that with more researches, measuring blood glucose level in suspected cardiac patients admitted to emergency departments can be used as a diagnostic and predictive tool for MACE.

***Limitations***

There were some limitations in the present study. Because of the large number of patients presenting to the emergency department of Imam-Hossein Hospital, assessing all suspected ACS patients was impossible and some eligible patients were probably missed. Fasting status of patients was unknown and thus the results may be skewed by patients that had recently consumed a high glucose load. The main goal of treatment staff of the hospital was secure treatment of cardiac patients and therefore, the patients were not under total control of researchers. ACS was diagnosed using common clinical judgments and atypical symptoms without chest discomfort were not used; this may result in missing some cases. Additionally, diabetic patients presenting with silent myocardial infarction were not included. In this study, three glucose groups were considered; however, since blood glucose is a continuous variable, a cut-off with optimum diagnostic and prognostic value in ACS should be found.

## Conclusion:

 It seems that abnormal admission blood glucose level in patients with suspected ACS was an independent predictor of major adverse cardiac events within 30 days. Yet, additional studies with greater sample sizes in emergency departments all over the country are required before it is applied in existing or future screening tools.
